# Deletion of pro-angiogenic factor vasohibin-2 ameliorates glomerular alterations in a mouse diabetic nephropathy model

**DOI:** 10.1371/journal.pone.0195779

**Published:** 2018-04-11

**Authors:** Kana Masuda, Katsuyuki Tanabe, Haruyo Ujike, Norikazu Hinamoto, Hiromasa Miyake, Satoshi Tanimura, Hitoshi Sugiyama, Yasufumi Sato, Yohei Maeshima, Jun Wada

**Affiliations:** 1 Department of Nephrology, Rheumatology, Endocrinology and Metabolism, Okayama University Graduate School of Medicine, Dentistry and Pharmaceutical Sciences, Okayama, Japan; 2 Department of Human Resource Development of Dialysis Therapy for Kidney Disease, Okayama University Graduate School of Medicine, Dentistry and Pharmaceutical Sciences, Okayama, Japan; 3 Department of Vascular Biology, Institute of Development, Aging, and Cancer, Tohoku University, Sendai, Japan; Hopital Tenon, FRANCE

## Abstract

Angiogenesis has been implicated in glomerular alterations in the early stage of diabetic nephropathy. We previously reported the renoprotective effects of vasohibin-1 (VASH1), which is a novel angiogenesis inhibitor derived from endothelial cells, on diabetic nephropathy progression. Vasohibin-2 (VASH2) was originally identified as a VASH1 homolog and possesses pro-angiogenic activity in contrast to VASH1. In addition, VASH2 was recently shown to promote epithelial-to-mesenchymal transition via enhanced transforming growth factor (TGF)-β signaling in cancer cells. Herein, we investigated the pathogenic roles of VASH2 in diabetic nephropathy using VAHS2-deficient mice. The type 1 diabetes model was induced by intraperitoneal injections of streptozotocin in VASH2 homozygous knockout (VASH2^*LacZ/LacZ*^) or wild-type mice. These mice were euthanized 16 weeks after inducing hyperglycemia. Increased urine albumin excretion and creatinine clearance observed in diabetic wild-type mice were significantly prevented in diabetic VASH2-deficient mice. Accordingly, diabetes-induced increase in glomerular volume and reduction in glomerular slit-diaphragm density were significantly improved in VASH2 knockout mice. Increased glomerular endothelial area was also suppressed in VASH2-deficient mice, in association with inhibition of enhanced vascular endothelial growth factor (VEGF) receptor 2 (VEGFR2), but not VEGF level. Furthermore, glomerular accumulation of mesangial matrix, including type IV collagen, and increased expression of TGF-β were improved in diabetic VASH2 knockout mice compared with diabetic wild-type mice. Based on the immunofluorescence findings, endogenous VASH2 localization in glomeruli was consistent with mesangial cells. Human mesangial cells (HMCs) were cultured under high glucose condition in *in vitro* experiments. Transfection of VASH2 small interfering RNA (siRNA) into the HMCs resulted in the suppression of type IV collagen production induced by high glucose compared with control siRNA. These results indicate that VASH2 may be involved in diabetes-induced glomerular alterations, particularly impaired filtration barrier and mesangial expansion. Therefore, VASH2 is likely to represent a promising therapeutic target for diabetic nephropathy.

## Introduction

Diabetic nephropathy is a leading cause of end-stage kidney disease (ESKD) in developed countries. In earlier stage, glomerular hyperfiltration, glomerular hypertrophy, glomerular basement membrane (GBM) thickening, and microalbuminuria are generally observed, followed by mesangial matrix expansion and proteinuria. Subsequently, nodular glomerulosclerosis and massive proteinuria develop in the advanced stage, leading to ESKD [1]. Despite advances in understanding the molecular mechanisms involving the development and progression of diabetic nephropathy, such as advanced glycation end-products, protein kinase C, and transforming growth factor-β (TGF-β) [2], certain effective therapeutic strategies remain to be established. Perhaps, multi-target therapy may be required for diabetic nephropathy treatment, and therefore, further identification of the potential therapeutic targets show great promise.

Angiogenesis, the growth of new blood vessels from pre-existing vessels, is associated with a number of pathological processes, and is also involved in the pathogenesis of diabetic nephropathy. Previous studies demonstrated new capillary formation and pre-existing capillary elongation [3, 4], as well as increased vascular endothelial growth factor (VEGF) level in diabetic glomeruli [5]. In addition, excessive activation of glomerular VEGF signaling in mice has been shown to cause mesangial matrix expansion, resembling diabetic nephropathy [6, 7]. Since the landmark study that revealed the renoprotective efficacy of anti-VEGF antibody in diabetic mice [8], anti-angiogenic strategies remain possible options for diabetic nephropathy treatment. In contrast, concerns regarding anti-VEGF antibody-induced renal thrombotic microangiopathy have limited anti-VEGF strategies [9]. Considering the possibility that anti-angiogenic strategies could suppress glomerular lesions in diabetes, including increased capillary area and mesangial expansion, novel angiogenic factors involved in the pathogenesis of diabetic nephropathy are likely to become promising therapeutic targets.

Vasohibin-1 (VASH1) is a unique endothelium-derived angiogenesis inhibitor, which prevents proliferation and migration of endothelial cells in an autocrine manner [10, 11]. We previously reported the therapeutic efficacy of adenoviral transfer of VASH1 in diabetic mice models [12, 13], and exacerbation of diabetic renal alterations in VASH1 heterozygous deficient mice [14], indicating the protective role of VASH1 in diabetic nephropathy. Vasohibin-2 (VASH2) was identified as a homolog to VASH1 [15]. In contrast to VASH1, VASH2 is known to possess pro-angiogenic activity [16]. Gene deletion of VASH2 or neutralizing antibody against it has been shown to inhibit cancer growth [17, 18]. Considering the above mentioned therapeutic effects of VASH1, VASH2 is expected to be a potential target for novel therapeutic strategy for diabetic nephropathy. Moreover, recent reports demonstrated that VASH2 could enhance TGF-β signaling in cancer cells [19]. Therefore, reduced VASH2 expression can possibly lead to the prevention of TGF-β-mediated glomerular alterations.

In the present study, we have demonstrated the improvement of diabetic nephropathy in VASH2-deficient mice, and the inhibition of high glucose-induced extracellular matrix (ECM) protein production in cultured mesangial cells with suppressed VASH2 expression.

## Materials and methods

### Animals and experimental protocols

**Fig 1:**
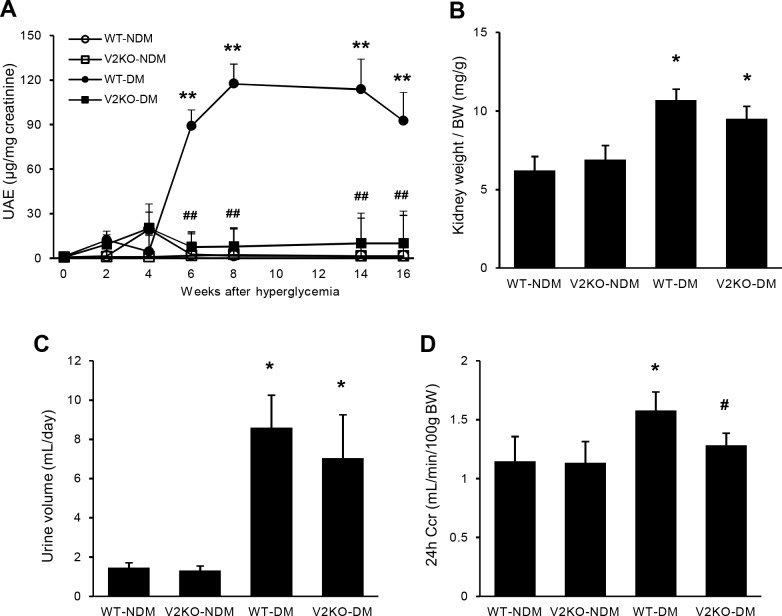
Urine albumin excretion, renal hypertrophy and creatinine clearance in non-diabetic and diabetic wild-type and VASH2 knockout mice. (A) Six weeks after the induction of hyperglycemia, albuminuria in diabetic wild-type (WT) mice (solid circles) was significantly exacerbated compared with that in non-diabetic WT mice (open circles). Although no difference was found in albuminuria between non-diabetic WT and non-diabetic VASH2 knockout mice (open squares), increased albuminuria induced by hyperglycemia was markedly prevented in diabetic VASH2 knockout mice (solid squares). (B, C) The increase in kidney weight-to-body weight ratio (B) and urine volume (C) induced by hyperglycemia did not significantly differ between WT and VASH2 knockout mice. (D) The increase in creatinine clearance (Ccr) level induced by hyperglycemia was significantly prevented in VASH2 knockout mice compared with WT mice. n = 6 for non-diabetic WT, 6 for non-diabetic VASH2 knockout, 10 for diabetic WT, and 8 for diabetic VASH2 knockout mice. *P<0.05 versus non-diabetic WT or VASH2 knockout mice, **P<0.01 versus non-diabetic WT or VASH2 knockout mice, ^#^*P*<0.05 versus diabetic WT mice, ^##^*P*<0.01 versus diabetic WT mice. Each column shows the mean ± SE.

**Fig 2:**
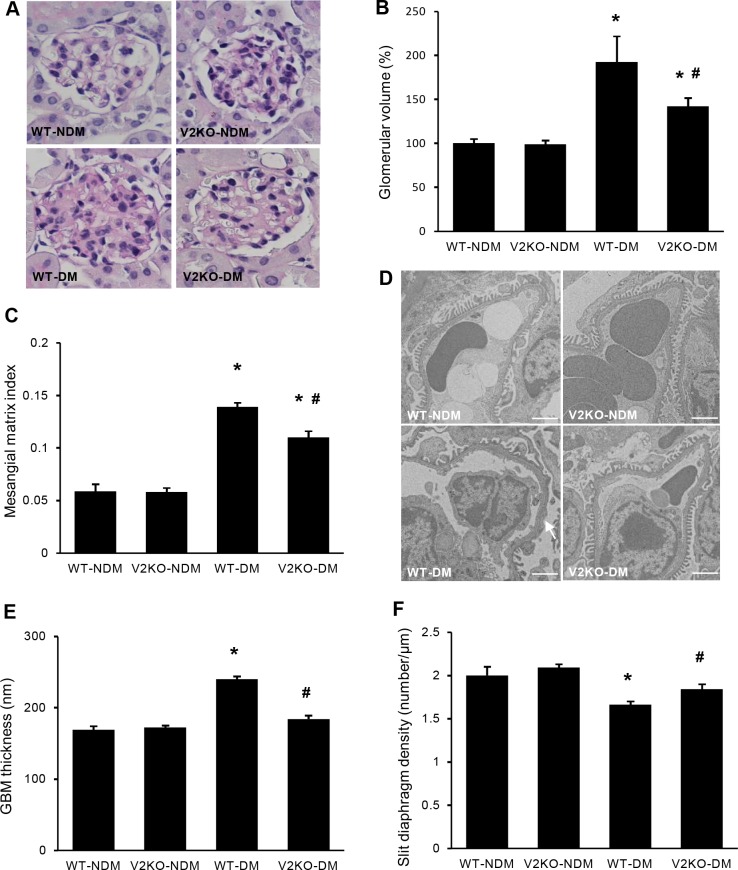
Histological and ultrastructural alterations in diabetic VASH2 knockout mice. (A) Representative light microscopic images of glomeruli from non-diabetic wild-type, non-diabetic VASH2 knockout, diabetic wild-type, and diabetic VASH2 knockout mice (periodic acid-Schiff staining, original magnification, ×400). (B, C) Diabetes-induced increases in glomerular volume (B) and mesangial matrix index (C) were significantly prevented in VASH2 knockout mice compared with wild-type mice. The mesangial matrix index was defined as the proportion of the glomerular volume occupied by mesangial matrix area (excluding nuclei). (D) Representative transmission electron microscopic images of glomerular capillary tufts from non-diabetic wild-type, non-diabetic VASH2 knockout, diabetic wild-type, and diabetic VASH2 knockout mice (scale bars, 2 μm). Diabetes caused foot process fusion (arrow) and obscured silt-diaphragms. (E) Increased GBM thickness in diabetic wild-type mice was significantly suppressed in diabetic VASH2 knockout mice. (F) The decrease in slit diaphragm density observed in diabetic wild-type mice was also improved in diabetic VASH2 knockout mice. n = 6 for non-diabetic WT, 6 for non-diabetic VASH2 knockout, 10 for diabetic WT, and 8 for diabetic VASH2 knockout mice. *P<0.05 versus non-diabetic WT or VASH2 knockout mice, ^#^*P*<0.05 versus diabetic WT mice. Each column shows the mean ± SE.

VASH-2 homozygous knockout (VASH-2^*LacZ/LacZ*^) mice were obtained from the Institute of Development, Aging, and Cancer, Tohoku University (Sendai, Japan). The experimental protocol was approved by the Animal Care and Use Committee in Okayama University (Okayama, Japan; approval number OKU-2014508). Male C57BL/6J wild-type (WT) and C57BL/6J VASH-2^*LacZ/LacZ*^ mice were fed a standard pellet laboratory chow and were provided with water *ad libitum*. Type 1 diabetes was induced by intraperitoneal injection of 50 mg/kg streptozotocin (STZ; Sigma-Aldrich, St Louis, MO) for five consecutive days [20, 21], as detailed by the NIDDK Consortium for Animal Models of Diabetic Complications protocol. Two weeks after the injection, the mice with hyperglycemia (defined as non-fasting blood glucose concentration of >15.5 mmol/l) were selected for experiments. Sixteen weeks after the selection, blood samples were collected from inferior vena cava, and the kidneys were harvested. All mice were euthanized using carbon dioxide gas. The experimental subgroups included (1) non-diabetic WT (WT-NDM), (2) non-diabetic VASH-2^*LacZ/LacZ*^ (V2KO-NDM), (3) diabetic WT (WT-DM) and (4) diabetic VASH-2^*LacZ/LacZ*^ (V2KO-DM) mice. In Table 1 and Figs 1 and 2, we used the following; six for WT-NDM, six for V2KO-NDM, ten for WT-DM and eight for V2KO-DM mice. However, we used six mice for each group in the remaining experiments.

**Table 1:** Characteristics of non-diabetic and diabetic wild-type and VASH2-deficient mice at the end of study.

	BW (g)	SBP (mmHg)	HbA1c (%)	sCr (mg/dl)
WT-NDM	32.2 ± 1.0	102.8 ± 4.2	4.0 ± 0.7	0.14 ± 0.06
V2KO-NDM	32.7 ± 0.9	111.7 ± 4.3	4.2 ± 0.7	0.16 ± 0.04
WT-DM	25.0 ± 0.7**	116.0 ± 3.0^#^	7.3 ± 0.5**	0.15 ± 0.03
V2KO-DM	26.1 ± 0.8**	112.7 ± 3.3	7.7 ± 0.6**	0.15 ± 0.04

BW, body weight; SBP, systolic blood pressure; HbA1c, hemoglobin A1c; sCr, serum creatinine; V2KO, VASH2 knockout; n = 6 for non-diabetic WT (WT-NDM), 6 for non-diabetic VASH2 knockout (V2KO-NDM), 10 for diabetic WT (WT-DM) and 8 for diabetic VASH2 knockout (V2KO-DM) mice. Data were expressed as mean ± SE.

**P<0.01 vs. WT-NDM group.

^#^*P*<0.05 vs. WT-NDM group.

### Blood and urine examination and blood pressure measurement

24 hours urine was collected in metabolic cages to evaluate urine albumin excretion, creatinine concentration, and daily urine volume. Blood glucose was measured in mouse tail-vein blood using a glucometer Glutest Neo (Sanwa Kagaku, Nagoya, Japan). Serum and urine creatinine levels, urine albumin concentration, and HbA1c were determined as previously described [22]. Creatinine clearance (Ccr) was calculated and expressed as milliliters per min per 100 g of body weight. Arterial blood pressure was measured by a programmable sphygmomanometer (BP-2000 Blood Pressure Analysis System for Mice and Rats; Visitech Systems Inc., Apex, NC) using the tail-cuff method as previously described [14].

### Histological analysis

10% buffered formalin-fixed and paraffin-embedded 4 μm sections were stained with periodic acid Schiff (PAS) for light microscopy observation. The mean glomerular volume (*G*_V_) was determined from the mean glomerular cross-sectional tuft area (*G*_A_), as previously described [22, 23]. Twenty glomerular images from each kidney section were taken, and the areas surrounded by glomerular capillary tufts were measured using Lumina Vision software (Mitani, Fukui, Japan) to determine the mean *G*_A_. *G*_V_ was calculated by the following equation:

$$G_{V}=\beta /k\times {\left(G_{A}\right)}^{3/2}$$

Here, β = 1.38 is the shape coefficient for spheres and *k* = 1.1 is a size distribution coefficient. *G*_V_ was expressed relative to the value of the WT-NDM group.

The mesangial matrix index was defined as the proportion of the mean *G*_A_ occupied by the mesangial matrix, excluding nuclei on PAS-stained specimens, as previously described [22, 23]. The mesangial matrix areas of twenty glomeruli in each kidney were selected using Photoshop software (Adobe Systems, San Jose, CA), followed by analysis using Lumina Vison software. Kidney sections were observed by two investigators (K.M. and H.U.) in a blinded manner.

### Immunofluorescence

Immunofluorescence was performed using frozen sections (4 μm), as previously described [24]. The following antibodies were used as primary antibodies: (1) monoclonal rat anti-CD31 antibody (BD Pharmingen, San Diego, CA); (2) polyclonal rabbit anti-type IV collagen antibody (Merck Millipore, Darmstadt, Germany); (3) monoclonal anti-β-galactosidase antibody (Promega, Madison, WI); (4) monoclonal rabbit anti-PDGFRβ antibody (Cell Signaling Technology, Danvers, MA); (5) polyclonal rabbit anti-ZO-1 antibody (Zymed, Carlsbad, CA). Alexa Fluor 488 or 546 (Thermo Fischer Scientific, Waltham, MA) was used as secondary antibodies. In double immunofluorescence, images were obtained using confocal laser microscopy (LSM780; Carl Zeiss, Oberkochen, Germany) in the Central Research Laboratory, Okayama University Medical School.

### Transmission electron microscopy

Transmission electron microscopy was performed as previously described [14]. The kidney tissues were fixed with 2.5% glutaraldehyde solution, postfixed in 1% osmium tetroxide, and then embedded in EPON epoxy resins. Ultra-thin sections were observed in a transmission electron microscopy (H-7650; Hitachi, Tokyo, Japan) in the Central Research Laboratory, Okayama University Medical School. GBM thickening was measured as the width of lamina densa at ten points per capillary tuft, with equal intervals, using Lumina Vison software. The slit diaphragm density was defined as number of open slit pores per unit length of capillary tuft. The length of capillary tuft was measured using Lumina Vison. Four capillary tufts in each mouse were assessed for the electron microscopic analyses and the data were averaged.

### Immunoblot analysis

Immunoblot assay was performed, as previously described [25]. (1) anti-VEGF-A antibody (Abcam, Cambridge, UK), (2) anti-VEGFR2 antibody (Cell Signaling), (3) anti-type IV collagen antibody (Abcam), (4) monoclonal anti-human VASH2 antibody (clone 1760; provided by Tohoku University, Sendai, Japan), and (5) monoclonal anti-β-actin antibody (Sigma-Aldrich) were used as primary antibodies. Among these, anti-type IV collagen antibody was used under non-reducing condition. HRP-conjugated anti-rabbit or mouse IgG antibodies (Cell Signaling) were served as secondary antibodies. Images were obtained with ImageQuant LAS 4000 (GE Healthcare, Pittsburgh PA).

### RNA extraction and real-time polymerase chain reaction

RNA extraction and real-time polymerase chain reaction (PCR) were performed, as previously described [12, 13], with modification. The following oligonucleotide primers were used: mouse TGF-β1, 5ʼ-GTG TGG AGC AAC ATG TGG AAC TCT A-3ʼ (forward) and 5’-TTG GTT CAG CCA CTG CCG TA-3ʼ (reverse); mouse VASH2, 5’-GGC TAA GCC TTC AAT TCC CC-3’ (forward) and 5’-CCC ATT GGT GAG ATA GAT GCC-3’ (reverse); mouse 18S rRNA, 5’-ACT CAA CAC GGG AAA CCT CA-3’ (forward) and 5’-AAC CAG ACA AAT CGC TCC AC-3’ (reverse); human VASH2, 5’-ACG TCT CAA AGA TGC TGA GG-3’ (forward) and 5’-TTC TCA CTT GGG TCG GAG AG-3’ (reverse); human type IV collagen α3, 5’-CAG GAC TCA CGG GTT CCA AAG-3’ (forward) and 5’-TGC ATC CTG GTA CAC CGA CAA-3’ (reverse); human connective tissue growth factor (CTGF), 5’- CTT GCG AAG CTG ACC TGG AA-3’ (forward) and 5’- AAA GCT CAA ACT TGA TAG GCT TGG A-3’ (reverse); human 18S rRNA, 5’-ACT CAA CAC GGG AAA CCT CA-3’ (forward) and 5’-AAC CAG ACA AAT CGC TCC AC-3’ (reverse). Real-time PCR amplifications were performed using SYBR Green PCR Master Mix (Applied Biosystems, Foster City, CA), the abovementioned primers and StepOnePlus™ Real-Time PCR System (Applied Biosystems).

### Cell culture

Human mesangial cells (purchased from Lonza, Basel, Switzerland) were cultured in Dulbecco's Modified Eagle's medium (DMEM; Sigma-Aldrich) supplemented with 10% fetal bovine serum, penicillin (100 U/ml) and streptomycin (100 μg/ml). For the experimental study, human mesangial cells were incubated in DMEM with normal glucose (NG; 5.5 mM) and high glucose (HG; 25 mM) for 24 hours after serum starvation. Normal glucose plus 19.5mM mannitol (MN) was used as osmotic control. For VASH2 gene-silencing study, cells were transfected with siRNA using Stealth Select RNAi (Invitrogen, Carlsbad, CA), as previously described [26]. The nucleotide sequences of human VASH2 siRNA were 5’-CAC UCU GAA UGA AGU GGG CUA UCA A-3’, and control siRNA was 5’-CGA CCU GCC CAA GAU UCC CAU ACC A-3’.

### Statistical analysis

All values are expressed as the mean ± standard error (SE). A Kruskal Wallis test with post-hoc comparisons using the Scheffe’s test was utilized for inter-group comparisons of multiple variables. The statistical analysis was performed using the JMP 10 software (SAS Institute Inc, Cary, NC). A level of *P* <0.05 was considered statistically significant.

## Results

### Improvement of urine albumin excretion and hyperfiltration in VASH2-deficient diabetic mice

STZ-induced diabetes resulted in significantly increased hemoglobin A1c (HbA1c) and, systolic blood pressure (SBP), and body weight (BW) loss, but did not change the serum creatinine (sCr) level. VASH2-deficiency did not affect BW, SBP, HbA1c, and sCr compared with WT mice. When diabetes was induced, no differences in these parameters were found between diabetic WT and VASH2-deficient mice (Table 1). In non-diabetic condition, VASH2 deficiency also had no effects on urinary albumin excretion (UAE), kidney weight/body weight (KW/BW) ratio, daily urine volume, and Ccr (Fig 1). Although diabetes led to marked increase in UAE in WT mice, it was significantly and almost entirely prevented in VASH2-deficient diabetic mice (Fig 1A). Despite no difference in increased KW/BW ratio and urine volume between diabetic WT and VASH2-deficient mice (Fig 1B and 1C), elevated Ccr level induced by diabetes in WT mice was significantly reduced in VASH2-deficient mice (Fig 1D), suggesting the potential improvement in diabetes-induced glomerular hyperfiltration with VASH2 deletion.

### Improvement of glomerular alterations in VASH2-deficient diabetic mice

No differences in glomerular histology between non-diabetic WT and VASH2-deficient mice were observed on light microscopy (Fig 2A). Diabetes-induced glomerular hypertrophy and mesangial expansion were significantly improved in VASH2-deficient diabetic mice (Fig 2A–2C). On electron microscopy, any ultrastructural changes of glomerular filtration barrier were not detected in both non-diabetic WT and VASH2-deficient mice (Fig 2D). GBM thickening and decreased slit-diaphragm density observed in diabetic WT mice were significantly prevented in VASH2-deficient mice (Fig 2D–2F). These results indicated that VASH2 may participate in the alterations of glomerular filtration barrier, leading to increased UAE.

### Improvement of glomerular endothelial alterations in VASH2-deficient diabetic mice

**Fig 3:**
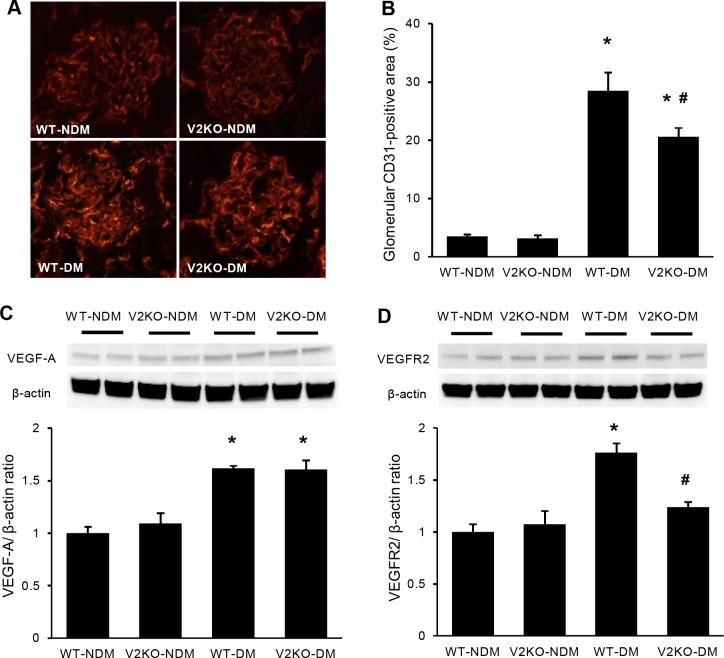
Alterations of glomerular endothelial area and VEGF-A expression in diabetic VASH2 knockout mice. (A) The distribution of CD31, a marker for endothelial cells, was determined by immunofluorescence in non-diabetic wild-type, non-diabetic VASH2 knockout, diabetic wild-type, and diabetic VASH2 knockout mice (original magnification, ×400). (B) In quantitative analysis, CD31-positive glomerular endothelial area was expanded in diabetic wild-type mice, but it was significantly prevented in diabetic VASH2 knockout mice. No difference was found in endothelial area between non-diabetic wild-type and non-diabetic VASH2 knockout mice. (C, D) Immunoblot for vascular endothelial growth factor-A (VEGF-A; C) and VEGF receptor-2 (VEGFR2; D). Each lane was loaded with 40 μg of protein obtained from the renal cortex. Each band was scanned and subjected to a densitometric analysis. Increased VEGF-A level induced by diabetes showed no difference between wild-type and VASH2 knockout mice, whereas increased VEGFR2 expression seen in diabetic wild-type mice was significantly suppressed in diabetic VASH2 knockout mice. n = 6 for each group. *P<0.05 versus non-diabetic WT or VASH2 knockout mice, ^#^*P*<0.05 versus diabetic WT mice. Each column shows the mean ± SE.

Considering previous reports that VASH2 directly acts on endothelial cells to exert proangiogenic properties [27], alterations of glomerular endothelial area were investigated. Glomerular capillary area detected by immunofluorescence of CD31, a marker of endothelial cell, was markedly increased in diabetic WT mice compared with non-diabetic WT mice. Although there was no difference in glomerular capillary area between non-diabetic WT and VASH2-deficient mice, diabetic VASH2-deficient mice showed significantly smaller capillary area than diabetic WT mice (Fig 3A and 3B). These results indicated that VASH2 deficiency did not decrease the basal glomerular capillary area but prevented the increased glomerular capillary area, corresponding to excessive angiogenic response in diabetic condition.

VEGF-A is essential for the integrity of glomerular capillary structure and function [28]. In diabetic glomeruli, upregulation of VEGF-A (produced by podocytes) and VEGF receptor 2 (VEGFR2; expressed on endothelial cells) has been demonstrated [12, 29]. In this study, no significant difference in renal VEGF-A and VEGFR2 levels was found between non-diabetic WT and VASH2-deficient mice (Fig 3C and 3D). Elevated level of renal VEGF-A induced by diabetes in WT mice was not affected by VASH2 deficiency (Fig 3C). However, enhanced VEGFR2 expression in diabetic WT mice was significantly prevented in diabetic VASH2-deficient mice (Fig 3D). Thus, VASH2 was likely to have direct effects on endothelial cells, rather than podocytes, in association with the impairment of glomerular filtration barrier.

### Improvement of glomerular collagen deposition in VASH2-deficient diabetic mice

**Fig 4:**
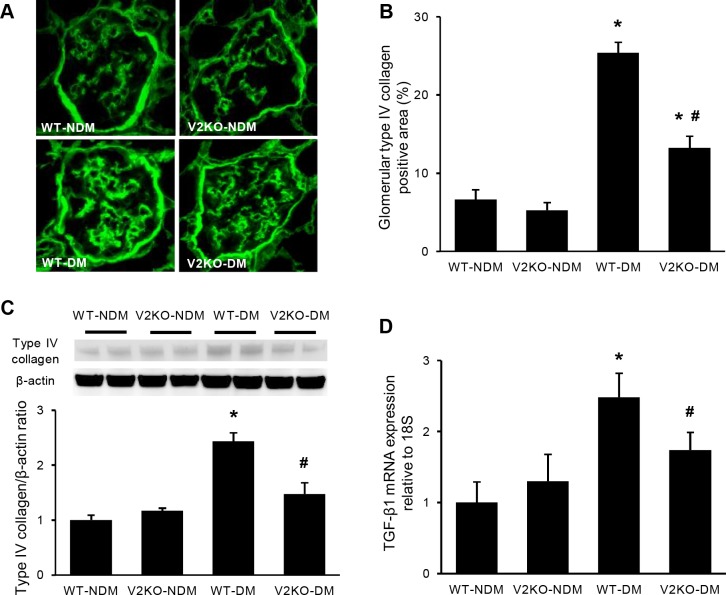
Mesangial matrix accumulation and TGF-β expression in diabetic VASH2 knockout mice. (A) The glomerular accumulation of type IV collagen was assessed by immunofluorescence for non-diabetic wild-type, non-diabetic VASH2 knockout, diabetic wild-type and diabetic VASH2 knockout mice (original magnification, ×400). (B) Immunoreactivity for type IV collagen was enhanced in diabetic wild-type mice compared with non-diabetic wild-type and VASH2 knockout mice. The accumulation of type IV collagen in glomeruli was prevented in diabetic VASH2 knockout mice. (C) Immunoblot for type IV collagen in renal cortex revealed the similar result to that of immunofluorescence study. Each lane was loaded with 40 μg of protein. Each band was scanned and subjected to a densitometric analysis. (D) Real-time PCR analysis for profibrotic factor, transforming growth factor-β (TGF-β) is shown. Increased level of TGF-β in diabetic wild-type mice was significantly prevented in diabetic VASH2 knockout mice. n = 6 for each group. *P<0.05 versus non-diabetic WT or VASH2 knockout mice, ^#^*P*<0.05 versus diabetic WT mice. Each column shows mean ± SE.

Consistent with glomerular mesangial expansion on light microscopy, diabetes induced prominent glomerular deposition of type IV collagen in WT mice (Fig 4A). Increased glomerular type IV collagen expression was significantly suppressed in diabetic VASH2-deficient mice (Fig 4A and 4B). Similarly, an immunoblot for type IV collagen demonstrated that increased expression of type IV collagen in diabetic kidneys was significantly prevented in diabetic VASH2 deficient mice (Fig 4C). TGF-β1 is well known to promote the production of ECM proteins by mesangial cells in a variety of glomerular diseases [30]. TGF-β1 expression in renal cortex was markedly increased in diabetic WT mice compared with non-diabetic WT and VASH2-deficient mice, consistent with previous reports [12, 13]. Such increased level of TGF-β1 level was significantly prevented in diabetic VASH2-deficient mice (Fig 4D).

### Glomerular expression of endogenous VASH2

**Fig 5:**
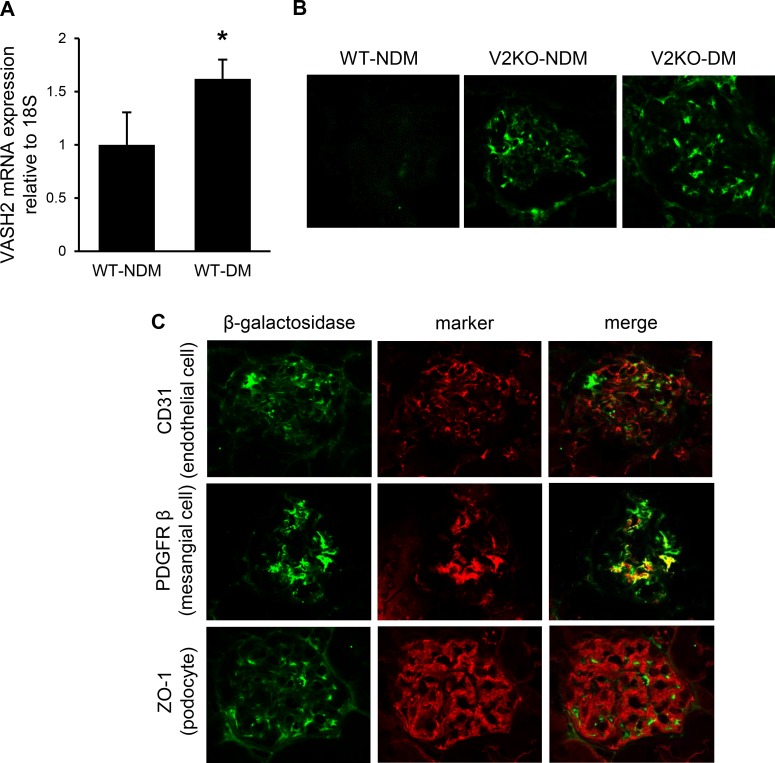
Localization of endogenous VASH2 in glomeruli. (A) The mRNA level of VASH2 in the kidney cortex from wild-type mice was assessed by real-time PCR. VASH2 expression was increased in diabetic condition compared with non-diabetic mice kidney. n = 6 for each group. *P<0.05 versus wild-type mice. (B) VASH2 expression is detected with immunofluorescence for β-galactosidase. No immunoreactivity in glomeruli is seen in wild-type mice (left panel), whereas glomeruli from non-diabetic and diabetic VAHS2 knockout (VASH2^*LacZ/LacZ*^) mice (middle and right panel, respectively) show a β-galactosidase-positive area (original magnification, ×400). (C) Double immunofluorescence for β-galactosidase and markers for glomerular component cells in VASH2 knockout mice show that the localization of β-galactosidase-positive area are consistent with platelet-derived growth factor receptor-β (PDGFRβ)-positive mesangial cells, but not CD31-positive endothelial cells and zonula occludens-1 (ZO-1)-positive podocytes.

Endogenous VASH2 mRNA expression in renal cortex was significantly up-regulated in diabetic mice (Fig 5A). Because β-galactosidase (β-gal) gene was inserted into the VASH2 knockout allele [16], we identified the VASH2 expression using β-gal staining in VASH2-deficient mice kidney. No immunoreactivity for β-gal was detected in glomeruli from WT mice kidney, whereas both non-diabetic and diabetic VASH2-deficient mice showed β-gal-positive area in glomeruli (Fig 5B). Double immunofluorescence study was performed in VASH2-deficient mice to clarify the localization of glomerular VASH2 expression. Immunoreactivity for β-gal consisted with platelet-derived growth factor receptor-β (PDGFRβ)-positive mesangial cell area, but not CD31-positive endothelial cell or zonula occludens-1 (ZO-1)-positive epithelial cell area, suggesting that endogenous VASH2 locally expressed in mesangial cells in glomeruli (Fig 5C).

### Role of VASH2 in extracellular matrix production by mesangial cells

**Fig 6:**
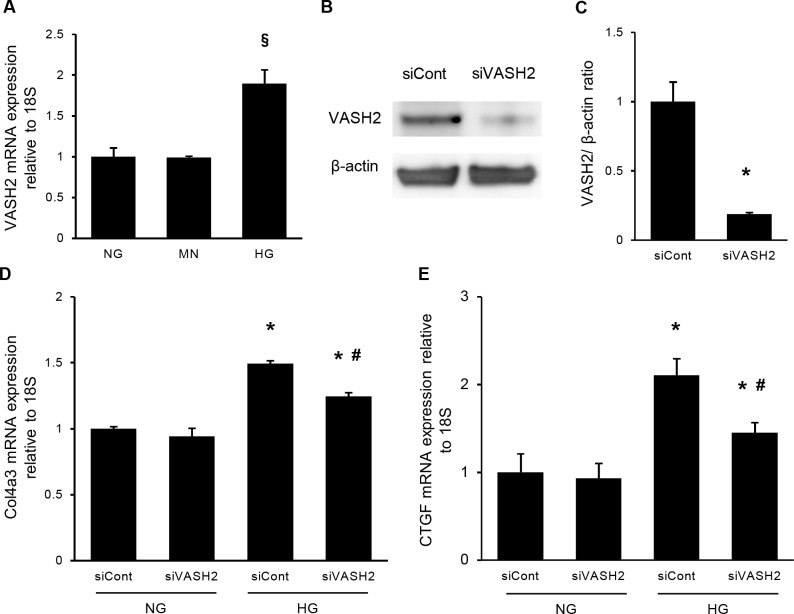
The role of VASH2 knockdown in extracellular matrix production in cultured mesangial cells. Human mesangial cells (HMCs) were cultured under normal glucose (NG, 5.5 mM), NG plus mannitol (MN; NG plus mannitol, 19.5 mM) or high glucose (HG, 25 mM) condition for 24 hours in the presence of negative control siRNA (siCont, 10 nM) or VASH2 siRNA (siVASH2, 10 nM). (A) The expression of VASH2 mRNA relative to 18S rRNA was elevated in HG but not in MN condition. (B, C) Immunoblots for VASH2 and β-actin are shown (B). Each lane was loaded with 20 μg of protein obtained from cultured HMCs. Each band was scanned and subjected to a densitometric analysis (C). Transfection of siVASH2 reduced the level of VASH2 by approximately 80%. (D, E) The amount of type IV collagen α3 (D) and connective tissue growth factor (CTGF; E) relative to 18S rRNA is shown. Increase in these mRNA level caused by HG condition was significantly prevented by transfection of siVASH2. n = 4 for each group. ^§^*P*<0.05 versus NG or MN, *P<0.05 versus NG with siCont or NG with siVASH2, ^#^*P*<0.05 versus HG with siCont. Each column shows the mean ± SE.

*In vitro* experiments using cultured human mesangial cells (HMCs) under normal glucose (NG) or high glucose (HG) condition were performed to clarify the potential role of VASH2 expressed in mesangial cells. The 24-hour incubation in HG condition significantly increased VASH2 mRNA expression in cultured HMCs compared with NG condition. However, addition of mannitol (MN) to NG condition could not induce the upregulation of VASH2 expression (Fig 6A). Transfection with VASH2 small interfering RNA (siRNA) decreased the levels of VASH2 protein by approximately 80% compared with the non-specific negative control siRNA (control siRNA) in HMCs under NG condition (Fig 6B and 6C). Increased expression of type IV collagen in HMCs under HG condition was observed (Fig 6D), as shown in previous reports [12, 22]. Incubation with VASH2 siRNA did not affect the collagen production by HMCs under NG condition, but significantly prevented it under HG condition (Fig 6D). The connective tissue growth factor (CTGF) is known to be implicated in ECM production in mesangial cells [31]. Higher expression of CTGF induced by HG condition was also suppressed by VASH2 knockdown (Fig 6E). Therefore, VASH2 expression in mesangial cells may accelerate glomerular extracellular matrix production observed in diabetic nephropathy.

## Discussion

In the present study, we revealed the improvement of urine albumin excretion and glomerular alterations in diabetic VASH2-deficient mice and the association between VASH2 expression and ECM production in cultured mesangial cells.

Human VASH2 was originally identified as a homolog of vasohibin-1 (VASH1), with 52.5% of the homology between the two protein sequences [15]. In contrast to anti-angiogenic activity of VASH1, VASH2 has been shown to possess pro-angiogenic activity and promote angiogenesis in various malignancies [32]. This protein is secreted from the producing cells, and acts on endothelial cells to promote its proliferation [27] and inhibit the termination of vessel sprouting [16]. VASH2 expression in cancer could be associated with the progression and poor clinical prognosis including pancreatic and breast carcinomas [33, 34]. However, the expression level of VASH2 is extremely low in differentiated cells except for cancer cells, whereas higher expression has been observed in embryonic stem (ES) cells and induced pluripotent stem (iPS) cells [35]. Any roles of VASH2 in non-neoplastic disorders remain to be elucidated.

One of the most notable findings in this study was the prevention of diabetes-induced urine albumin excretion in VASH2-deficient mice. A unique crosstalk between endothelial cells and podocytes has been recognized to maintain integrity of glomerular filtration barrier [9, 28]. In the glomeruli, VEGF-A is produced by and secreted from podocytes, and acts on endothelial cells to keep their structure and function. However, increased VEGF induced by diabetes could not only increase the number of glomerular capillaries, but also enhance glomerular endothelial permeability, leading to the disruption of the filtration barrier and subsequent albuminuria [9]. Diabetes-induced endothelial dysfunction may accelerate such glomerular abnormalities [36]. In this study, VAHS2 deficiency prevented the increased glomerular capillary area in diabetic mice. However, VASH2 deficiency did not affect VEGF-A level in both non-diabetic and diabetic conditions, whereas increased VEGFR-2 expression induced by diabetes was suppressed in diabetic VASH2-deficient mice, suggesting that VASH2 might enhance VEGF signaling in endothelial cells. In addition, another study demonstrated that VASH2 expression did not correlate with VEGF-A level in gastric cancer [37]. Thus, VASH2 deficiency may not inhibit excessive VEGF-A production. Recent clinical evidence, which suggests that anti-VEGF monoclonal antibody for treatment of metastatic cancer causes proteinuria and renal thrombotic microangiopathy, has dampened the enthusiasm for anti-angiogenic strategies for diabetic nephropathy. Traditional anti-angiogenic therapy that directly targets VEGF-A has a potential concern for promoting endothelial injury, since endogenous VEGF-A is required for maintaining endothelial integrity. However, VASH2 deficiency did not deplete VEGF-A in either non-diabetic or diabetic conditions, as shown in Fig 3C. In addition, given that VASH2 knockout mice have no obvious phenotype, VASH2-targeting therapy for diabetic nephropathy is unlikely to result in the glomerular endothelial injury that was observed in anti-VEGF antibody-treated patients. Unfortunately, receptor(s) and intracellular signaling pathways for VASH2 in endothelial cells have not been clarified yet. Improvement of podocyte damage observed in diabetic VASH2-deficient mice is likely to relate with reduced urine albumin excretion. Because our previous study demonstrated the direct effects of VASH1 not only on glomerular endothelial cells but also on podocytes [12, 13], whether VASH2 directly affects podocyte integrity should be investigated in future studies.

Mesangial expansion is a characteristic glomerular alteration in diabetic nephropathy, leading to glomerulosclerosis [21]. Mesangial cells are stimulated by hyperglycemia combined with TGF-β1 in diabetes to obtain fibroblast-like properties characterized by the expression of α-smooth muscle actin (SMA) and production of ECM proteins. Anti-TGF-β antibody suppressed ECM gene expression in diabetic animals [38]. In this study, VASH2 was localized in glomerular mesangial cells and its deficiency resulted in the attenuation of mesangial collagen accumulation and TGF-β1 expression in diabetic mice. A recent report suggested that up-regulation of VASH2 resulted in epithelial to mesenchymal transition (EMT) of cancer cells [39]. More recently, VASH2 derived from cancer cells was shown to stimulate migration of and α-SMA expression in fibroblasts [40]. Therefore, VASH2 might accelerate diabetes-induced transformation of mesangial cells to fibroblast-like cells. In addition, our *in vitro* experiment demonstrated that knockdown of VASH2 in mesangial cells prevented the increased mRNA level of type IV collagen induced by high glucose concentration. VASH2 has been reported to increase the expression of TGF-β type I receptor and promote the downstream signaling [19]. These results suggest that VASH2 would be a novel target of TGF-β-dependent pathological processes, including diabetic nephropathy.

As described above, no specific phenotype in non-diabetic condition was observed in VASH2-deficient mice compared with WT mice, consistent with other previous reports [16, 17]. Therefore, VASH2 expression is probably not essential for maintaining physiological angiogenesis. Because VEGF inhibition often leads to proteinuria and renal thrombotic microangiopathy [41], VASH2 is likely to be a superior target for anti-angiogenic therapy in diabetic nephropathy, similar to cancers [18]. However, VASH2 deficiency was shown to inhibit hypoxia-mediated subcutaneous angiogenesis [16], and thus, VASH2-targeting therapy might impair some normal angiogenesis, such as wound healing. Further investigations should be needed to address such concerns.

This study has several limitations. First, we evaluated the role of endogenous VASH2 in a type 1 diabetes model, and thus, the use of type 2 diabetes model should be considered in the future. Second, as we utilized the traditional (but not conditional) gene knockout mice in this study, whether our results might be ascribed to circulating VASH2 or renal VASH2 remains unclear. Considering up-regulation of VASH2 in cultured mesangial cells treated with high glucose concentration, renal VASH2 should play significant roles in diabetic nephropathy. However, further experiments using conditionally knocked out VASH2 mice are warranted.

## Conclusion

In conclusion, endogenous VASH2 may exacerbate urine albumin excretion and mesangial expansion in diabetic nephropathy, possibly through enhancing VEGFR-2 signaling in glomerular endothelial cells and ECM production in mesangial cells, thus indicating that VASH2 could represent a potential therapeutic target for diabetic nephropathy.
